# Enhanced Inflammatory Potential of CD4^+^ T-Cells That Lack Proteasome Immunosubunit Expression, in a T-Cell Transfer-Based Colitis Model

**DOI:** 10.1371/journal.pone.0095378

**Published:** 2014-04-16

**Authors:** Orhan Rasid, Chantal Meulenbroeks, Andrea Gröne, Dietmar Zaiss, Alice Sijts

**Affiliations:** 1 Department of Infectious Diseases and Immunology, Faculty of Veterinary Medicine, University of Utrecht, Utrecht, The Netherlands; 2 Department of Pathology, Faculty of Veterinary Medicine, University of Utrecht, Utrecht, The Netherlands; French National Centre for Scientific Research, France

## Abstract

Proteasomes play a fundamental role in intracellular protein degradation and therewith regulate a variety of cellular processes. Exposure of cells to (pro)inflammatory cytokines upregulates the expression of three inducible catalytic proteasome subunits, the immunosubunits, which incorporate into newly assembled proteasome complexes and alter the catalytic activity of the cellular proteasome population. Single gene-deficient mice lacking one of the three immunosubunits are resistant to dextran sulfate sodium (DSS)-induced colitis development and, likewise, inhibition of one single immunosubunit protects mice against the development of DSS-induced colitis. The observed diminished disease susceptibility has been attributed to altered cytokine production and CD4^+^ T-cell differentiation in the absence of immunosubunits. To further test whether the catalytic activity conferred by immunosubunits plays an essential role in CD4^+^ T-cell function and to distinguish between the role of immunosubunits in effector T-cells versus inflamed tissue, we used a T-cell transfer-induced colitis model. Naïve *wt* or immunosubunit-deficient CD4^+^ T-cells were adoptively transferred into RAG1^−/−^ and immunosubunit-deficient RAG1^−/−^ mice and colitis development was determined six weeks later. While immunosubunit expression in recipient mice had no effect on colitis development, transferred immunosubunit-deficient T- cells were more potent in inducing colitis and produced more proinflammatory IL17 than *wt* T-cells. Taken together, our data show that modifications in proteasome-mediated proteolysis in T-cells, conferred by lack of immunosubunit incorporation, do not attenuate but enhance CD4^+^ T-cell-induced inflammation.

## Introduction

The immune system senses pathogens through pattern recognition receptors that bind specific pathogen-associated molecular patterns. Ligand binding induces a signaling cascade downstream of the receptor that activates a specific transcriptional program, allowing the immune system to respond efficiently to the invading microorganisms. The proteasome, an abundant cellular protease complex, plays an essential role in those signaling pathways, as the activation of many signaling molecules is regulated by the timely degradation of other molecules in the signaling complex. So depends the activation of the transcription factor NFκB on phosphorylation, ubiquitylation and subsequent proteasome-mediated degradation of its inhibitor IκB [1]. IκB degradation exposes a nuclear localization sequence in NFκB, allowing it to translocate to the nucleus and to initiate the expression of, amongst others, (pro)inflammatory cytokines [1]–[3]. Another function of proteasomes, during infection with intracellular pathogens, is the processing of pathogen-derived antigens into peptides that can be presented by MHC class I molecules on the cell surface, allowing CD8 T-cells to detect and react to the presence of intracellular pathogens (for review see [4]). Thus, proteasome activity plays an essential role at different stages of pathogen-specific immune responses.

Proteasomes consist of a barrel-shaped catalytic core particle, the 20S proteasome, and one or more regulatory particles (for review see [5]). The enzymatic activity of the 20S proteasomes is exerted by three β subunits, located in the inner two rings of the 20S complex, which exhibit caspase-like (β1), trypsin-like (β2) and chymotrypsin-like activity (β5). Exposure of cells to type 1 and type 2 interferons or TNFα induces the expression of three facultative subunits, β1i/LMP2, β2i/MECL-1 and β5i/LMP7, which preferentially incorporate into newly assembled proteasome complexes and thus, when expressed, replace their constitutive homologues in the cellular proteasome population [5]. In addition, in particular cells of the hematopoietic lineage express different quantities of the three facultative subunits and, therefore, often contain so called “mixed” proteasomes, containing the constitutive and one or more inducible β subunits [5], [6]. Due to altered cleavage preferences, proteasomes containing the facultative subunits (named immunoproteasomes) are more suited to generate high affinity MHC class I ligands than constitutive proteasomes, containing the β1, β2 and β5 subunits [5], [7], [8]. As a consequence, pathogen-specific CD8^+^ T-cell responses often target immunoproteasome-generated peptides [5], [8], [9]. Immunoproteasomes have further been shown to protect cells from interferon-induced oxidative stress, by efficient removal of aggregates of oxydant-damaged, polyubiquitylated unfolded nascent proteins [10], [11] and immunoproteasome expression in the peripheral tissues was found to protect against early forms of CD8^+^ T-cell-mediated autoimmune diseases [7], [10]. In specific, we showed that irradiated and BM reconstituted, β2i/MECL-1&β5i/LMP7-deficient recipient mice developed latent forms of CD8^+^ T-cell-mediated autoimmune diseases, such as insulin-dependent diabetes mellitus and diabetes insipidus [7]. This could be explained by altered MHC class I antigen processing of tissue antigens in inflamed immunosubunit-deficient tissue, in particular, since adoptive transfer of CD4^+^ T-cell depleted splenocytes from diseased mice conferred the disease phenotype onto RAG1^−/−^ mice that also lacked immunosubunit expression, but not onto RAG1^−/−^ mice that expressed the immunosubunits [7]. Alternatively, enhanced death in immunosubunit-deficient tissues following irradiation [10] may have led to presentation of tissue antigens in the draining lymph nodes and thus may have induced priming of auto-reactive CD8^+^ T-cells.

In hematopoetic cells, expression of the immunosubunits supports the production of many cytokines. [12]–[14]. Based on the latter observations, immunoproteasomes have been proposed to drive inflammatory processes, an assumption that is supported further by the anti-inflammatory effects of proteasome subunit-specific inhibitors [14]. The role of immunoproteasomes in inflammatory diseases has been investigated in further detail in different mouse models of colitis [15]–[17]. These studies showed that inflammatory disease was accompanied by high levels of immunosubunit expression in the inflamed tissue, with cellular infiltration and expression of inflammatory cytokines [15], [16]. Inhibition of activity of the proteasome subunit LMP7/β5i or of both LMP7/β5i and β5 largely prevented DSS-induced colitis, and was associated with reduced quantities of inflammatory cytokines [14]–[16]. Notably, a deficiency of β5i/LMP7, in mice lacking the gene encoding this subunit, protected from DSS-induced colitis [15], [16]. This was attributed to diminished processing of the transcription factor NFκB, which initiates cytokine transcription, in hematopoietic cells by Schmidt et al. [15]. Groettrup and coworkers [17], however, reported a reduced differentiation of “proinflammatory” Th1 and Th17 cells and enhanced differentiation of regulatory T-cells (Tregs) in β5i/LMP7^−/−^ mice, suggesting that altered T-cell differentiation may explain for the ameliorating effects of β5i/LMP7 deficiency in development of DSS-induced colitis. The last authors also showed that LPS or anti-CD3 stimulated spleen cells of *wt* and β5i/LMP7^−/−^ mice produce similar amounts of IL-6, IFNy and IL-17 [14], [16], arguing against a direct effect of LMP7 on the production of those cytokines. Taken together, these data establish an important regulatory role for the immunosubunit β5i/LMP7 in inflammatory disease, but the underlying molecular mechanism remains poorly understood. In addition, the protective effects of β5i/LMP7 deficiency in inflammatory disease are remarkable in light of the observed importance of immunoproteasomes in clearing potentially toxic protein aggregates in cytokine-exposed cells [10].

To further investigate the role of proteasome immunosubunits in autoimmune, inflammatory disease, we analyzed T-cell-induced colitis development in RAG1 gene-deficient mice compared to RAG1&β2i/MECL-1&β5i/LMP7-deficient mice, in a T-cell transfer model. Using this model, we were able to distinguish between disease modifying effects of immunosubunit expression in inflamed tissue and in effector T-cells, respectively.

## Results

### β2i/MECL-1&β5i/LMP7 Deficiency does not Protect against T-cell Transfer-induced Colitis

**Figure 1:**
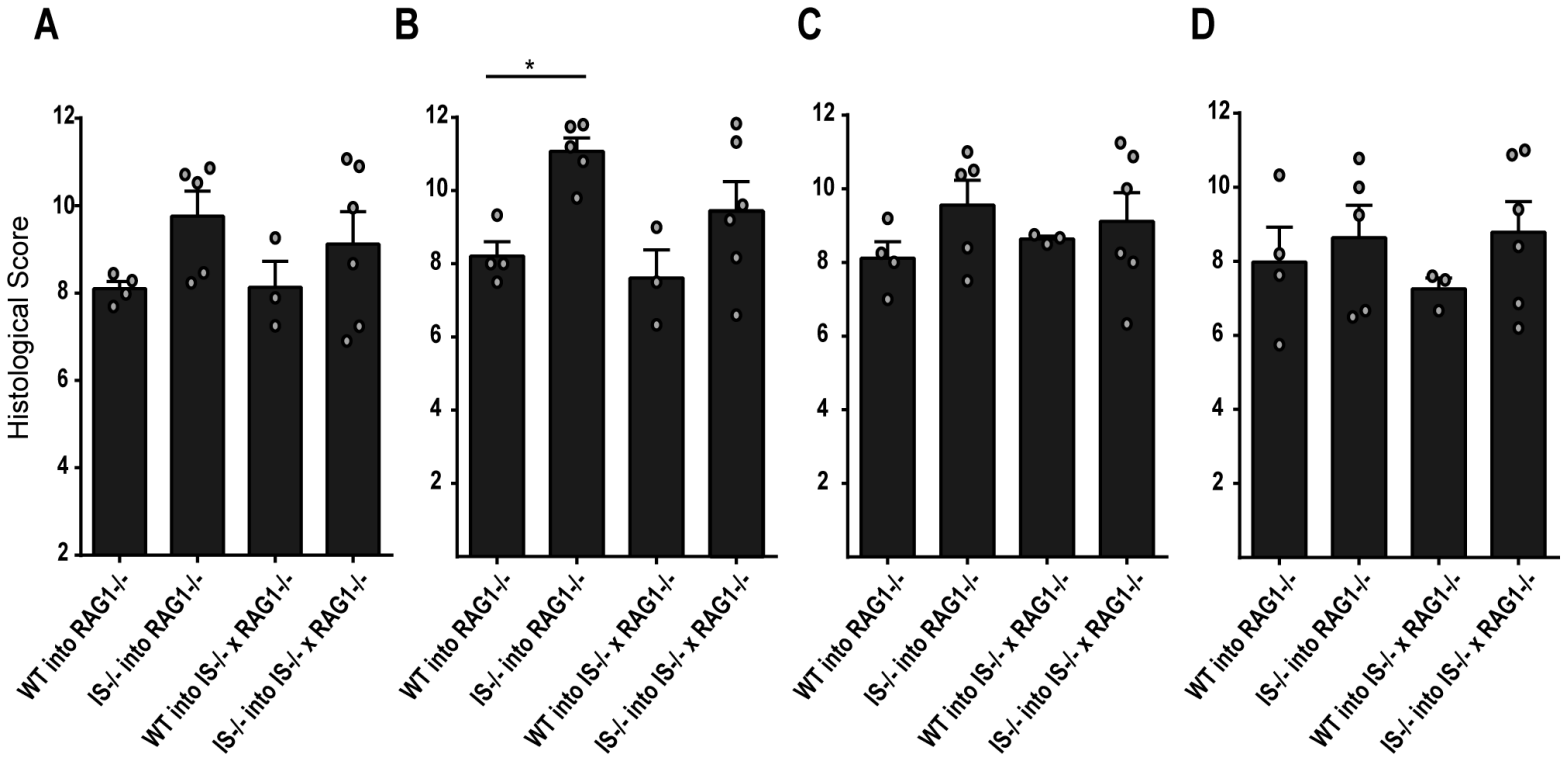
Colitis development in T-cell transferred mice. Flow cytometry-sorted naïve CD4^+^ T-cells of B6 (WT) or β2i/MECL-1^−/−^β5i/LMP7^−/−^(IS−/−) mice were transferred into RAG1^−/−^ or RAG1^−/−^β2i/MECL-1^−/−^β5i/LMP7^−/−^ (IS−/− x RAG1−/−) mice and colitis development was determined 6 weeks by histological scoring of H&E stained tissue samples (see M&M). (A) overall colitis scores, (B) colitis scores in the proximal −, (C) mid −, and (D) distal colon sections. Scores for individual mice and means+SEM (n = 3–6 per group) are depicted. *p<0.05. Data are representative of two independent experiments.

Previous studies have shown that the absence or the inhibition of the immunosubunit β5i/LMP7 protects mice against development of DSS-induced colitis, which was attributed to diminished transcription of (pro)inflammatory cytokines or to diminished Th1 and Th17 and enhanced Treg cell differentiation [15], [17]. To determine to which extent the absence of immunosubunit expression in effector T-cells versus in inflamed tissue confers resistance to colitis development, we employed a well-established T-cell transfer-induced colitis model [18]–[20]. Purified naïve *wt* or β2i/MECL-1&β5i/LMP7–deficient T-cells were transferred into RAG1-gene-deficient mice or β2i/MECL-1&β5i/LMP7 deficient mice that had been backcrossed onto the RAG1^−/−^ background. Six weeks after cell transfer, mice were sacrificed and colitis development was determined by histological scoring (for criteria, see Materials and Methods). As expected, RAG-1^−/−^ mice transferred with *wt* CD4^+^ T-cells developed colitis (Figure 1A, Figure S1) and so did transferred RAG1^−/−^β2i/MECL-1^−/−^β5i/LMP7^−/−^ mice, with similar severity as RAG1^−/−^mice. Surprisingly, β2i/MECL-1^−/−^β5i/LMP7^−/−^ CD4^+^ T-cells also caused colitis, both in RAG1^−/−^ and RAG1^−/−^β2i/MECL-1^−/−^β5i/LMP^−/−^ mice (Figure 1A, Figure S1). Histological scores in mice that received β2i/MECL-1&β5i/LMP7-deficient T-cells were even higher than in recipients of *wt* CD4^+^ T-cells (Figure 1B–D, Figure S1), with significant differences in the proximal colon (Figure 1B, Figure S1). Thus, neither a lack of proteasome immunosubunit expression in effector T-cells nor in the inflamed tissue conferred resistance to development of T-cell transfer-induced colitis.

**Figure 2:**
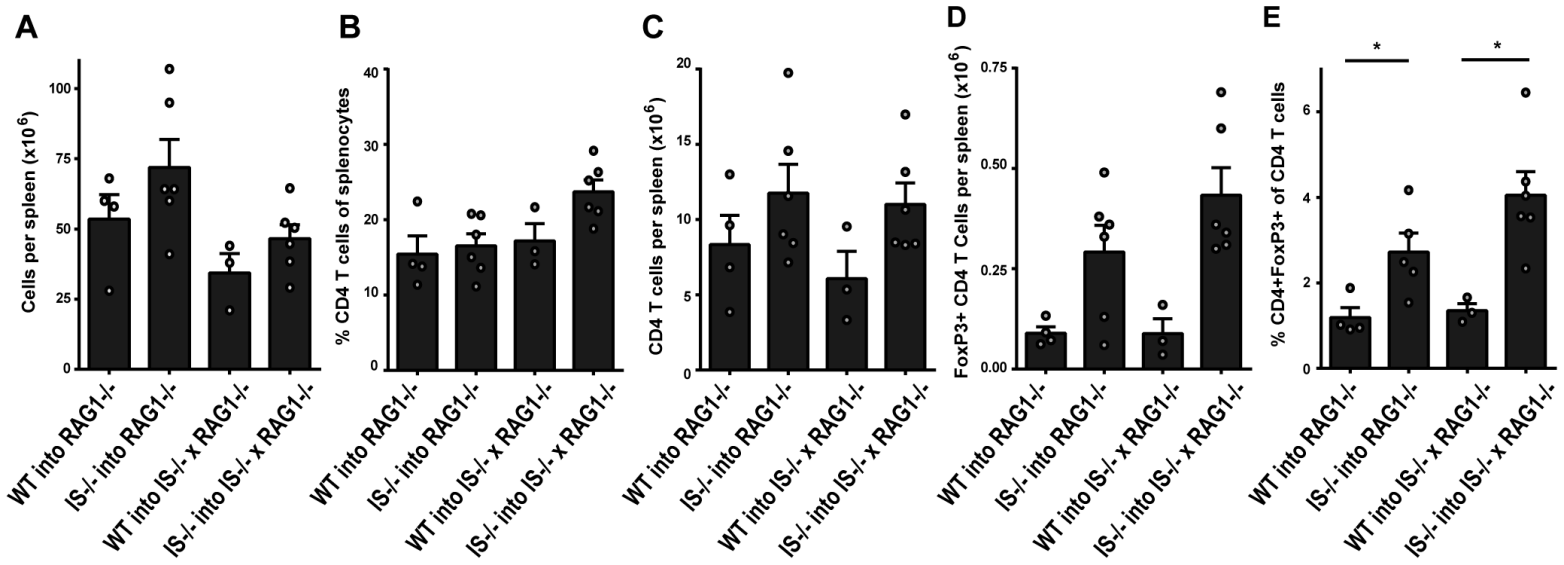
CD4 T cells and iTreg cells in mice with T-cell transfer-induced colitis. Splenocytes of mice with T-cell transfer-induced colitis, harvested six weeks after T-cell transfer, were stained for CD4 and FoxP3 expression and analyzed by flow cytometry. (A) absolute numbers of splenocytes; (B, D) percentages, and (C, E) numbers of CD4+ T-cells and CD4+FoxP3+ T-cells in the spleen. Results for individual mice and means+SEM (n = 3–6 per group) are depicted. *p<0.05.

To determine how colitis development in RAG1^−/−^ mice and RAG1^−/−^β2i/MECL-1^−/−^β5i/LMP7^−/−^ mice correlated with expansion of transferred, naïve CD4^+^ T-cells, spleens of these mice were harvested and analyzed. Total numbers of splenocytes were lower in transferred RAG1^−/−^β2i/MECL-1^−/−^β5i/LMP7^−/−^ mice than in transferred RAG1^−/−^ mice (Figure 2A), consistent with the reduced spleen size and numbers of total splenocytes often observed in β2i/MECL-1&β5i/LMP7-deficient compared to *wt* mice (D.Z. and A.S., unpublished observations). Analysis of the splenic CD4^+^ T-cell populations by flow cytometry revealed no major differences in percentages of CD4^+^ T-cells between the different mouse groups (Figure 2B), but total numbers of CD4^+^ T-cells were higher in mice transferred with β2i/MECL-1&β5i/LMP7-deficient CD4^+^ T-cells than in recipients of *wt* CD4^+^ T-cells (Figure 2C), which was in direct correlation with the enhanced severity of inflammation in the colon (Figure 1). These data show that β2i/MECL-1&β5i/LMP7-deficiency does not impair the proliferative potential of CD4^+^ T-cells.

### Enhanced Induced (i)Treg Differentiation and Elevated Levels of IL17 in Colitic Mice that Received β2i/MECL-1&β5i/LMP7-deficient CD4^+^ T-cells

β5i/LMP7-deficient mice are resistant to development of DSS-induced colitis, due to reduced NFκB activation or to altered CD4^+^ T-cell differentiation, which both may dampen the production of (pro)inflammatory cytokines [15], [17]. Intracellular staining of FoxP3 in spleen-derived CD4^+^ T-cells of mice with T-cell transfer-induced colitis (Figure 2D, E) showed enhanced numbers and percentages of FoxP3^pos^ cells, i.e. iTregs, in mice that had received β2i/MECL-1&β5i/LMP7-deficient CD4^+^ T-cells. On the other hand, deficiency of β2i/MECL-1&β5i/LMP7 in recipient mice had no effect on iTreg differentiation (Figure 2D, E).

**Figure 3:**
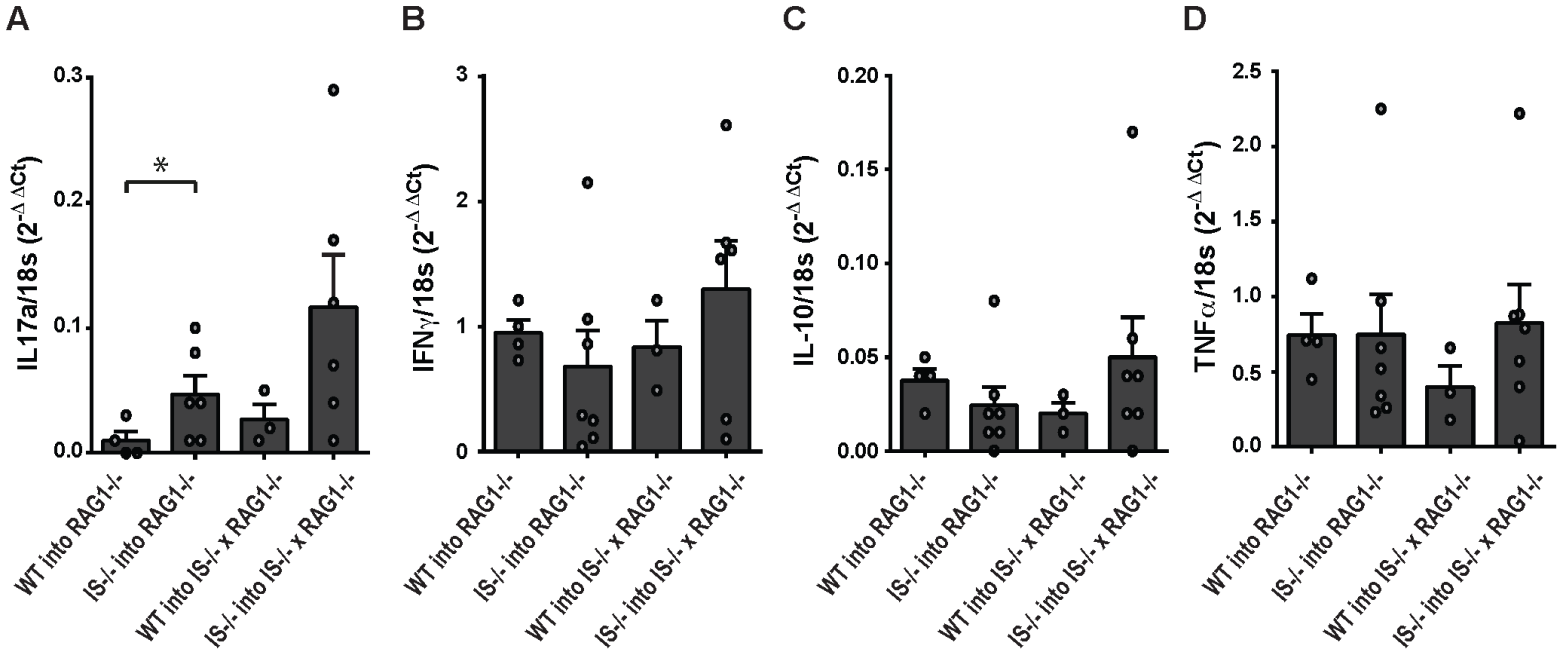
Cytokine expression in mice with T-cell transfer-induced colitis. Splenocytes of mice with T -cell transfer-induced colitis were stimulated with anti-CD3 mAb for 4 hrs. mRNA was extracted and cytokine expression was quantified by RT-PCR. Expression of (A) IL17a, (B) IFNy, (C) IL10, and (D) TNFα relative to 18S rRNA is shown for individual mice per experimental group. Bars represent means+SEM (n = 3–6). *p<0.05. Data are representative of two independent experiments.

Analysis of cytokine expression in TCR-activated splenic T-cells by RT-PCR demonstrated enhanced quantities of IL17 mRNA in recipients of β2i/MECL-1&β5i/LMP7-deficient CD4^+^ T-cells (Figure 3A), while expression levels of IFNy, IL10 and TNFα did not differ between the different mouse groups (Figure 3B–D). Thus, both iTregs and IL17-producing cells were more frequent in spleens of colitic mice that had been transferred with β2i/MECL-1&β5i/LMP7-deficient CD4^+^ T-cells than in mice that had received *wt* CD4^+^ T-cells.

## Discussion

Previous studies have shown that proteasome immunosubunits may modify host susceptibility to autoimmune and inflammatory diseases at multiple levels, i.e. by altering MHC class I antigen processing in inflamed tissues, by modulating the ability of cells to cope with cell stress and by modulating signal transduction and transcriptional programs in cells, leading to modified T-cell differentiation [7], [10], [15], [17]. We have used a murine model of T-cell transfer-induced colitis to further examine the role of the proteasome immunosubunits in inflammatory diseases. We show that naïve CD4^+^ T-cells of mice that lack the two immunosubunits β2i/MECL-1&β5i/LMP7 cause colitis upon transfer into RAG1^−/−^ mice, which is more severe than colitis induced by transfer of naïve *wt* CD4^+^ T-cells. Enhanced colitis scores were associated with enhanced numbers of total splenic CD4^+^ T-cells, enhanced frequencies and numbers of iTregs and enhanced IL17 production. Previous studies in humans and DSS-treated mice showed that not only immune cells but also inflamed colon tissue expressed the proteasome immunosubunits [16], [21]. In our experiments, the severity of T-cell transfer-induced colitis did not differ between Rag1^−/−^ mice and RAG1^−/−^ mice that had been backcrossed onto an β2i/MECL-1&β5i/LMP7-deficient background (Figure 1), arguing against a role for immunosubunit expression in the inflamed gut in colitis development. This is consistent with prior observations, which showed that transplantation of *wt* bone marrow rendered β5i/LMP7-deficient mice susceptible to development of DSS-induced colitis [15]. Thus, in the T-cell transfer model, β2i/MECL-1&β5i/LMP7-deficiency in transferred, naïve CD4^+^ T-cells enhances colitis development in RAG1^−/−^ mice, whereas immunosubunit-deficiency in the targeted gut does not have any effect on colitis development.

Studies by different groups have demonstrated that proteasomes play an important role in cytokine production and that modulation of proteasome subunit composition may alter cytokine production. For example, dendritic cells of LMP2-deficient mice produce less IFNα, IL-6, IL-1β and TNFα following influenza virus infection than *wt* dendritic cells [12]. This defect was attributed to the formation of mixed proteasomes, containing the immunosubunits β2i/MECL-1 and β5i/LMP7 and the constitutive subunit β1/δ, which may diminish transcription of these cytokines by delaying NFκB processing in those cells. A diminished transcription of the same cytokines, IL17 and IL23 was detected also during DSS-induced colitis in β5i/LMP7-deficient, in β1i/LMP2 deficient- and in β2i/MECL-1-deficient mice, as well as in mice treated with selective inhibitors targeting the β5i/LMP7 and β5 subunits or the β5i/LMP7 subunit only [15], [16]. This was accompanied by a reduced activation of NFκB, in β5i/LMP7-deficient cells [15] and reduced Th1 and Th17 but enhanced Treg cell differentiation of CD4^+^ T-cells in the lamina propria of β5i LMP7-deficient/inhibitor-treated mice with mild forms of DSS-induced colitis [15]–[17]. Following T-cell transfer into RAG2^−/−^ mice, Kalim et al. [17] further observed a diminished Th1 and Th17 and enhanced Treg cell differentiation in recipients of β5i/LMP7-deficient CD4^+^ T-cells, suggesting that differences in T-cell differentiation explain for the relative resistance of inhibitor-treated or β5i/LMP7-deficient mice to development of DSS-induced colitis.

In agreement with Kalim et al [17], we observe an enhanced differentiation of naïve immunosubunit-deficient CD4^+^ T-cells into iTregs in adoptively transferred RAG1^−/−^ mice. However, we also find that these iTregs fail to protect the transferred mice against colitis development. Thus, recipients of β2i/MECL-1&β5i/LMP7-deficient T-cells develop colitis six weeks after T-cell transfer, with even higher histological scores and higher quantities of IL17α in the spleens than detected in recipients of *wt* CD4^+^ T-cells, despite enhanced numbers and frequencies of iTregs. On the other hand, FACS-purified Tregs derived from β2i/MECL-1&β5i/LMP7-deficient, immunocompetent mice suppress CD4^+^ T-cell-induced colitis upon cotransfer (not shown), indicating that β2i/MECL-1&β5i/LMP7-deficiency does not disable Treg function. Interestingly, in a recent publication, Basler et al. [22] showed that both β5i/LMP7-deficient and *wt* mice develop experimental autoimmune encephalitis (EAE) following immunization with the MOG_35–55_ peptide. Disease intensity did not significantly differ between mouse groups in this study, but was found to be more intense in β5i/LMP7-deficient mice by Seifert et al. [10], using the same model. As Th17 cells play a central role in the onset and maintenance of EAE, these studies indicate that an exchange of β5i/LMP7 for β5 in T-cells does not diminish T cell-induced inflammatory disease. Moreover, specific inhibition of β5i/LMP7 ameliorated peptide-immunization-induced EAE in *wt* mice, while specific inhibition of the constitutive proteasome subunit β5 ameliorated EAE in LMP7-deficient mice [22], indicating that the enzymatic activity of the β5/β5i proteasome active site subunit is critical for CD4^+^ T-cell function. Taken together, we conclude that the exchange of β5i/LMP7 for β5 in T cells does not abolish but may modulate CD4^+^ T-cell mediated disease, with aggravating effects on inflammation in case of T-cell transfer-induced colitis.

While our data show that immunosubunit-deficiency does not ameliorate T-cell transfer-induced colitis, immunosubunit-deficient mice are protected from DSS-induced colitis [15]–[17]. Although unlikely, it cannot be ruled out that this discrepancy between effects in the two models may be explained by the use of different immunosubunit deficient mice in these studies. While resistance to DSS-induced colitis was observed in single immunosubunit-deficient mice, lacking either β1i/LMP2, β2i/MECL-1 or β5i/LMP7 [15]–[17], we used β2i/MECL-1&β5i/LMP7-deficient T-cells to determine the effects of immunosubunit deficiency in development of T-cell transfer colitis. Thus we cannot exclude that specific proteasome subtypes [23], [24], present in single immunosubunit-deficient cells but lacking in double immunosubunit-deficient cells, or the other way around, are responsible for the discrepancy between effects of immunosubunit deficiency in colitis induction in the two models. A more likely explanation however lies in the involvement of different immune effector cells in DSS-induced compared to T-cell transfer-induced colitis and perhaps the differing roles of NFκB activation in these cell types. In DSS-induced colitis, inflammation is driven primarily by leukocytes of the innate immune system, such as monocyte-derived macrophages, which are dependent on NFκB activation for cytokine expression. Thus, delayed NFκB processing may modify the cytokine environment in DSS-treated mice and thereby prevent the onset of lead to modest inflammation at most. In T-cell transfer-induced colitis, inflammation is mediated by activated CD4^+^ T-cells. In T cells, NFκB activation plays a role only during priming, since CD28-mediated costimulatory signals are dependent on the NFκB pathway but once primed, T-cells secrete cytokines mainly upon antigen-induced TCR stimulation. As discussed above, β5i/LMP7-deficient T cells are activated in inflammatory disease models (as well as infection models) and secrete cytokines upon TCR engagement with quantities comparable to those secreted by *wt* T cells [14], [16], thus a possibly delayed NFκB activation does not impair their function.

Taken together, we conclude that immunosubunit deficiency may influence disease processes in different ways, depending on the etiology of disease. Our data argue against any direct attenuating effects on inflammatory CD4^+^ T-cells but, in contrary, show that β2i/MECL-1&β5i/LMP7-deficiency enhances CD4^+^ T-cell-mediated inflammation in T-cell transfer-induced colitis.

## Materials and Methods

### Mice

C57BL/6 (B6), B6 RAG-1^−/−^, B6 β5i/LMP7^−/−^β2i/MECL-1^−/−^ [9], [25], [26] and B6 RAG-1^−/−^β5i/LMP7^−/−^β2i/MECL-1^−/−^ mice [24] were maintained by in-house breeding under specific pathogen-free conditions or under standard conditions, in filter top cages. All experiments were performed with age-matched mice.

### Ethics Statement

All animal experiments were carried out in strict accordance with the Dutch Animal Experimentation Act and EU directives 86/609/CEE and 2010/63/EU related to the protection of vertebrate animals used for experimental or other scientific purposes. The experimental protocols were approved by the Committee on Animal Experiments of the University of Utrecht (DEC 2012.II.02.029) and performed in the Central Laboratory Animal Research Facility of the University of Utrecht, which has AAALAC (Association for Assessment and Accreditation of Laboratory Animal Care) accreditation.

### T-cell Transfer Colitis Model

To induce colitis, CD4^+^ CD45RB^high^ cells were FASC-purified from the spleens of B6 *wt* and B6 β2i/MECL-1^−/−^β5i/LMP7^−/−^ donor mice, using a BD Influx™ (BD Biosciences). A total of 4×10^5^ CD4^+^CD45RB^high^ cells were injected intraperitoneally (*i.p.*) in 200 µl PBS into B6 RAG-1^−/−^ or B6 RAG-1^−/−^β5i/LMP7^−/−^β2i/MECL-1^−/−^ recipient mice. Mice were monitored for 6 weeks for clinical signs and body weight and then sacrificed. Colons were flushed with 10% formalin to remove feces, fixed and stored in 70% ethanol for H&E histopathology. Colitis development was evaluated by histology by two independent experts in a blinded fashion, with minor modifications from the protocol described in [20]. In brief, inflammatory infiltrates, depletion of goblet cells and epithelial hyperplasia were scored as specified in Table 1. Scores for specific colon sections represent the sum of scores for these different criteria. The overall histological score per mouse colon is the average of individual scores for the proximal, mid and distal colon segments.

**Table 1:** Histological scoring of T-cell transfer colitis.

Score	Criteria
	**Inflammatory infiltrate**
0	No infiltration of mononuclear cells
1	Focal infiltration of mononuclear cells in the lamina propria
2	Multiple foci of mononuclear cells in the lamina propria
3	Multifocal infiltration distending the lamina propria and/or few cells in the submucosa
4	Evident infiltrate in the lamina propria and submucosa, distending the submucosal space
5	Severe transmural infiltration of mononuclear cells
	**Depletion of goblet cells**
0	No loss of goblet cells
1	Focal loss of goblet cells
2	Multifocal loss of goblet cells, but with areas with normal appearance
3	General diminished numbers of goblet cells
4	Depletion of goblet cells
	**Epithelial hyperplasia**
0	Normal epithelium
1	Few areas of mild hyperplasia
2	Mild epithelial hyperplasia
3	Severe hyperplasia with crowding of epithelial crypts

### Flow Cytometry and T-cell Stimulation

Spleens were passed through a 70 µm cell strainer to prepare single cell suspensions, and erythrocytes were lysed by treatment with ammonium chloride. Cells for T-cell transfer were stained with anti-CD4 mAb (RM4-5, eBioScience) in the presence of Fc-block, enriched using a BD Influx™ (BD Biosciences) and then were stained with anti-CD45RB (16A, BD Pharmingen) and anti-CD25 (PC61, eBioscience) mAbs and resorted to obtain a CD4^+^CD45RB^high^CD25^−^ population that was used for induction of colitis. To analyze spleens of T-cell transferred mice, total cell counts in single cell suspensions were determined and cell samples then were stained for CD4 cell surface and intracellular FoxP3 expression (clone FJK-16S, eBioscience) according to manufacturer’s instructions. Cells were measured on a FACSCantoII (BD Biosciences) and analyzed with FlowJo software (Tree Star). To determine cytokine expression, splenocytes were stimulated with or without anti-CD3 mAb (145.2C11, BD Pharmingen, 2 µg/ml) for 4 h, pelleted and then stored at −20°C in TRIzol reagent (Invitrogen, Breda, NL) prior to RNA purification.

### Quantitative PCR

RNA was extracted from TRIzol stored samples as instructed (Invitrogen, Breda, NL) and quantified using a Nanodrop ND-1000 (Thermo Scientific, Etten-Leur, NL). 1 µg of total RNA was reverse transcribed into cDNA synthesis using an iScript cDNA Synthesis Kit (Bio-Rad Laboratories, Veenendaal, NL). TaqMan® Gene Expression Assays (Applied Biosystems, Austin, TX, USA) were used for qRT-PCR amplification and expression of the following genes was assessed: IL17α, IFNy, TNFα and IL10. Relative expression of target genes was calculated using the Pfaffl method with 18S rRNA as a reference gene.

### Statistical Analysis

Statistical analyses were performed with GraphPad Prism 4.00 (Graphpad Software, San Diego, CA) using Mann–Whitney U test (+) to compare results between experimental groups. Significant results are at P≤0.05.

## Supporting Information

Figure S1 **Representative histological colon sections.** Flow cytometry-sorted naïve CD4^+^ T-cells of B6 (WT) or β2i/MECL-1^−/−^β5i/LMP7^−/−^ (IS−/−) mice were transferred into RAG1^−/−^ or RAG1^−/−^β2i/MECL-1^−/−^β5i/LMP7^−/−^ (IS−/− x RAG1−/−) mice and colitis development was determined 6 weeks later by histological scoring of H&E stained tissue samples (see Materials & Methods). Pictures were taken at 40x magnification. (A) Infiltrate in the lamina propria and submucosa with multifocal loss of goblet cells and mild epithelial hyperplasia; (B) Infiltrate in the lamina propria and submucosa with focal loss of goblet cells and mild epithelial hyperplasia; (C) Multifocal infiltration in the lamina propria with diminished number of goblet cells and mild epithelial hyperplasia; (D) Evident infiltration distending submucosal splace with depletion of goblet cells and severe epithelial hyperplasia with crowding of mucosal crypts; (E) Transmural infiltration with depletion of goblet cells and severe hyperplasia; (F) Evident infiltration with loss of goblet cells and severe epithelial hyperplasia; (G) Evident infiltration with multifocal loss of goblet cells and few areas of epithelial hyperplasia; (H) Multifocal infiltration in the lamina propria and loss of goblet cells with severe hyperplasia and crowding of crypts; (I) Multiple foci of inflammatory infiltrate and loss of goblet cells and mild hyperplasia. (J) Transmural infiltration with diminished goblet cells and mild hyperplasia; (K) Evident infiltration with depletion of goblet cells and severe hyperplasia; (L) Evident infiltrate in the lamina propria and submucosa with diminished goblet cells and hyperplasia. Data are representative of two independent experiments.
(PDF)
