# Supplementary figures and images for: Enhanced Inflammatory Potential of CD4+ T-Cells That Lack Proteasome Immunosubunit Expression, in a T-Cell Transfer-Based Colitis Model
Source: PLoS One. 2014 Apr 16;9(4):e95378. doi: 10.1371/journal.pone.0095378 (PMC3989320; doi:10.1371/journal.pone.0095378)

Figure S1

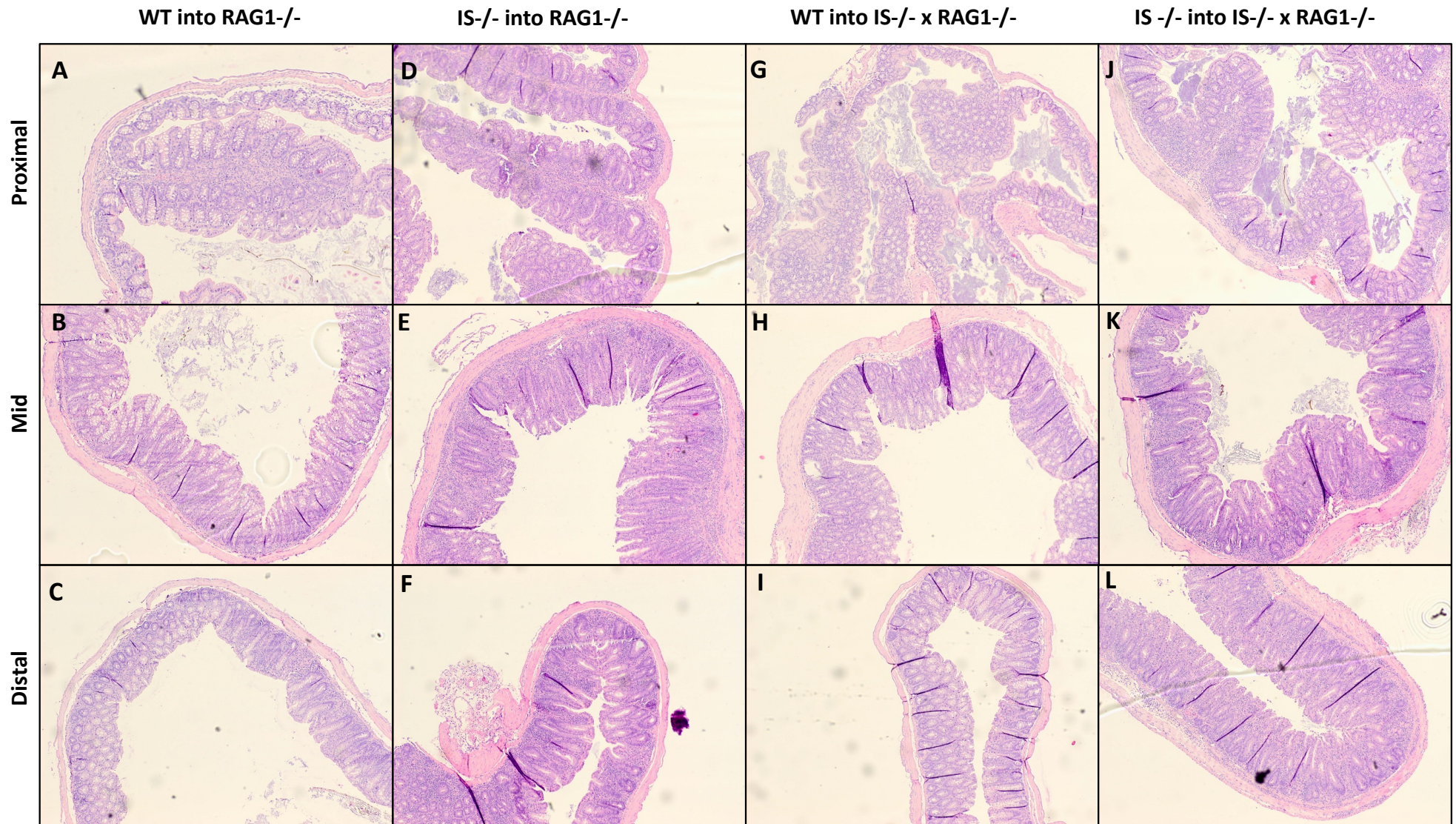

Supplement: Figure S1 — Representative histological colon sections. Flow cytometry-sorted naïve CD4+ T-cells of B6 (WT) or β2i/MECL-1−/−β5i/LMP7−/− (IS−/−) mice were transferred into RAG1−/− or RAG1−/−β2i/MECL-1−/−β5i/LMP7−/− (IS−/− x RAG1−/−) mice and colitis development was determined 6 weeks later by histological scoring of H&E stained tissue samples (see Materials & Methods). Pictures were taken at 40x magnification. (A) Infiltrate in the lamina propria and submucosa with multifocal loss of goblet cells and mild epithelial hyperplasia; (B) Infiltrate in the lamina propria and submucosa with focal loss of goblet cells and mild epithelial hyperplasia; (C) Multifocal infiltration in the lamina propria with diminished number of goblet cells and mild epithelial hyperplasia; (D) Evident infiltration distending submucosal splace with depletion of goblet cells and severe epithelial hyperplasia with crowding of mucosal crypts; (E) Transmural infiltration with depletion of goblet cells and severe hyperplasia; (F) Evident infiltration with loss of goblet cells and severe epithelial hyperplasia; (G) Evident infiltration with multifocal loss of goblet cells and few areas of epithelial hyperplasia; (H) Multifocal infiltration in the lamina propria and loss of goblet cells with severe hyperplasia and crowding of crypts; (I) Multiple foci of inflammatory infiltrate and loss of goblet cells and mild hyperplasia. (J) Transmural infiltration with diminished goblet cells and mild hyperplasia; (K) Evident infiltration with depletion of goblet cells and severe hyperplasia; (L) Evident infiltrate in the lamina propria and submucosa with diminished goblet cells and hyperplasia. Data are representative of two independent experiments. (PDF) [file pone.0095378.s001.pdf]
